# Assessing the contributions of childhood maltreatment subtypes and depression case-control status on telomere length reveals a specific role of physical neglect

**DOI:** 10.1016/j.jad.2017.01.031

**Published:** 2017-04-15

**Authors:** John Vincent, Iiris Hovatta, Souci Frissa, Laura Goodwin, Matthew Hotopf, Stephani L. Hatch, Gerome Breen, Timothy R. Powell

**Affiliations:** a King's College London, MRC Social, Genetic and Developmental Psychiatry Centre, Institute of Psychiatry, Psychology & Neuroscience, London, UK; b Department of Biosciences, University of Helsinki, Helsinki, Finland; c King's College London, Health Service & Population Research, Institute of Psychiatry, Psychology & Neuroscience, London, UK; d King's College London, Psychological Medicine, Institute of Psychiatry, Psychology & Neuroscience, London, UK; e University of Liverpool, Department of Psychological Sciences, Liverpool, UK; f National Institute for Health Research Biomedical Research Centre for Mental Health, Institute of Psychiatry, Psychology and Neuroscience at the Maudsley Hospital and King's College London, UK

## Abstract

**Background:**

Studies have provided evidence that both childhood maltreatment and depressive disorders are associated with shortened telomere lengths. However, as childhood maltreatment is a risk factor for depression, it remains unclear whether this may be driving shortened telomere lengths observed amongst depressed patients. Furthermore, it's unclear if the effects of maltreatment on telomere length shortening are more pervasive amongst depressed patients relative to controls, and consequently whether biological ageing may contribute to depression's pathophysiology. The current study assesses the effects of childhood maltreatment, depression case/control status, and the interactive effect of both childhood maltreatment and depression case/control status on relative telomere length (RTL).

**Method:**

DNA samples from 80 depressed subjects and 100 control subjects were utilized from a U.K. sample (ages 20–84), with childhood trauma questionnaire data available for all participants. RTL was quantified using quantitative polymerase chain reactions. Univariate linear regression analyses were used to assess the effects of depression status, childhood maltreatment and depression by childhood maltreatment interactions on RTL. The false discovery rate (q<0.05) was used for multiple testing correction.

**Results:**

Analysis of depression case/control status showed no significant main effect on RTL. Four subtypes of childhood maltreatment also demonstrated no significant main effect on RTL, however a history of physical neglect did significantly predict shorter RTL in adulthood (F(1, 174)=7.559, p=0.007, q=0.042, Variance Explained=4.2%), which was independent of case/control status. RTL was further predicted by severity of physical neglect, with the greatest differences observed in older maltreated individuals (>50 years old). There were no significant depression case/control status by childhood maltreatment interactions.

**Limitations:**

A relatively small sample limited our power to detect interaction effects, and we were unable to consider depression chronicity or recurrence.

**Conclusion:**

Shortened RTL was specifically associated with childhood physical neglect, but not the other subtypes of maltreatment or depression case/control status. Our results suggest that the telomere-eroding effects of physical neglect may represent a biological mechanism important in increasing risk for ageing-related disorders. As physical neglect is more frequent amongst depressed cases generally, it may also represent a confounding factor driving previous associations between shorter RTL and depression case status.

## Highlights

• Depression casesness, childhood emotional abuse, physical abuse, sexual abuse and emotional neglect did not predict shorter telomere length in our U.K. sample.
• Childhood physical neglect specifically predicted shortened telomere lengths, and this effect was independent from depression case-control status.
• Due to its higher frequency amongst depressed patients, physical neglect may represent one factor driving previous associations between shortened telomere lengths and depression.

## 1 Introduction

Psychiatric disorders, such as depression, have been linked to a heightened risk of severe medical conditions as well as early naturally occurring mortality (Viron and Stern, 2010; O'Donovan et al., 2011; Penninx et al., 2013). Medical conditions that are particularly frequent amongst depressive disorder patients tend to be those associated with ageing, including, but not limited to, cardiovascular disease, stroke, obesity, and type II diabetes (Evans et al., 2005). This has raised the question as to whether depression could be associated with accelerated biological ageing, i.e., when the ageing at the cellular level exceeds that of which is expected by chronological age (Lindqvist et al., 2015).

Indeed, advanced biological ageing has been demonstrated at the molecular level amongst depressed patients relative to controls in some previous reports (Hartmann et al., 2010). Telomeres are capping structures of repeat DNA at the end of chromosomes which act as biomarkers for cellular ageing (Eitan et al., 2014). Although there have been mixed reports, most individually conducted studies have shown that depressed patients exhibit shortened telomere lengths in their blood cells relative to controls, which is indicative of biologically older cells (Hartmann et al., 2010; Simon et al., 2006; Garcia-Rizo et al., 2013; Elvsåshagen et al., 2011; Hoen et al., 2011; Lung et al., 2007; Verhoeven et al., 2014; Wikgren et al., 2012). This association has further been confirmed in recent meta-analyses (Schutte and Malouff, 2015, Ridout et al., 2016, Darrow et al., 2016, Lin et al., 2016). If this ageing effect is causal, shortened telomeres may represent a mechanism contributing to the pathophysiology of depressive disorders, or if it represents a consequence of having depression, ageing cells may moderate the increased risk of comorbid ageing-related disorders reported previously.

Interestingly, childhood maltreatment has also been associated with shortened telomere lengths in adulthood, and it acts as a significant risk factor for depressive disorders (Tyrka et al., 2010; Kananen et al., 2010; O'Donovan et al., 2011; Shalev et al., 2014). What remains relatively unclear is whether childhood maltreatment could be driving the differences in telomere length previously observed amongst depressed cases. Alternatively, there could be depression case/control by maltreatment interactions, which moderate telomere length. For instance, if telomere lengths were found to be shorter amongst maltreated individuals, but with an even greater shortening amongst those with a subsequent depression diagnosis, it may suggest that shortened telomeres (and potentially advanced biological ageing) is a causal mechanism relating to depression.

The current study investigated relative telomere length (RTL) in a sample of 180 UK subjects (80 depressive disorder cases, 100 control subjects). Through the inclusion of maltreatment data and depression case/control data in the same analysis we aimed to disentangle each factor's contribution to biological ageing and to detect disease by maltreatment interactions.

## 2 Methods

### 2.1 Participants and diagnosis

A cohort study design assessing both depressive disorder cases and controls was implemented to address the research questions. A total of 180 White subjects from the United Kingdom were included in the study; 77 males and 103 females with ages ranging between 20 and 84 years (mean age of 50 (15.65 S.D)). This consisted of 80 DNA samples obtained from subjects with a depressive disorder and 100 DNA samples obtained from control individuals, see Table 1. All of the control subjects and the majority of depressive disorder case samples were obtained from the South East London Community Health Study (SELCoH; Brown et al., 2014, Hatch et al., 2011, Hatch et al., 2016). However, due to low numbers of depressive disorder cases, a further 25 case samples from the Depression Case Control Study (DeCC) were also included (Gaysina et al., 2007). Inclusion of subject samples for the current study was based on: (i) availability of leukocyte DNA samples, (ii) participants being White and from the UK (due to population differences in TL), (iii) availability of information on depressive disorder case/control status and childhood maltreatment.

**Table 1:** Table showing characteristics of our depressed cases and controls, including number of males, age, BMI and the number of mild/mixed/moderate-severe depressed cases.

	**No. of males (%)**	**Age (S.D.)**	**BMI (S.D.)**	**Mild depression**	**Moderate or severe depression**	**Mixed depression/anxiety**	**Total**
Controls	49 (49%)	50.84 (16.89)	26.89 (5.39)	–	–	–	100
Cases	28 (35%)	48.63 (13.97)	28.47 (6.87)	15	55	10	80

Control subjects were recruited as part of SELCoH and identified as having no current depressive disorder symptoms using the Clinical Interview Schedule-Revised (CIS-R) (Lewis et al., 1992), and no previous diagnoses of a depressive disorder, as assessed using a self-report questionnaire. Depressive disorder case status was characterised in SELCoH using the CIS-R, which uses an algorithm to approximate ICD-10 diagnoses (World Health Organisation, 1993). A subject was screened positive for a depressive disorder if they met diagnostic criteria for any of the following: mild depressive episode, mixed anxiety and depressive disorder, moderate/severe depressive episode. Although we considered ‘depressive disorder’ as a single group, for sensitivity analyses we did consider each of these groups separately. Depressive disorder diagnosis in DeCC was made using the Schedules for Clinical Assessment in Neuropsychiatry (SCAN), as described previously, with all DeCC subjects representing major depressive disorder patients, i.e. those with moderate/severe depressive episodes (Wing et al., 1998, Gaysina et al., 2007).

Data regarding age, gender, depressive disorder disease status, and maltreatment status was obtained from all participants, and body mass index (BMI) data was available for 98% of subjects. For SELCoH subjects only, we also had information regarding smoking habits, antidepressant use, drug dependency, drug use, other medication use, and comorbid diseases, allowing us to perform sensitivity analyses to test for potential confounders.

### 2.2 Assessment of childhood maltreatment

The presence or absence of childhood maltreatment or maltreatment subtypes (emotional abuse, physical abuse, sexual abuse, emotional neglect, and physical neglect) was determined using the Childhood Trauma Questionnaire (CTQ) (Bernstein et al., 2003). Due to relatively small sample sizes and fewer instances of severe maltreatment, for our primary analyses, we dichotomised subjects into those with, or without, a history of childhood maltreatment. To achieve this, we first determined those with no history of maltreatment, and those with mild, moderate and severe maltreatment, as according to Bernstein et al., 2003. We then collapsed the mild/moderate/severe maltreatment groups into a single maltreatment category. For secondary analyses, where we do find a significant effect of a maltreatment subtype, we further consider the impact of severity (no/mild/moderate-severe maltreatment).

### 2.3 Ethics

The SELCoH study received approval from the King's College London research ethics committee, reference PNM/12/13-152. The DeCC study was approved by the Joint South London and Maudsley NHS Trust Institute of Psychiatry Research Ethics Committee. Informed written consent was obtained from all the participants at the time of sample collection.

### 2.4 DNA extraction and telomere assessment

10 mL of blood was collected from subjects in tubes containing EDTA (BD Vacutainer; BD, NJ, USA) and stored at −80 °C. DNA was then extracted using a standard in-house protocol described previously (Freeman et al., 2003) and stored at −80 °C. All samples had 260/280 ratios of between 1.7 and 1.9 as tested using the Nanodrop D1000 (Thermoscientific, Wilmington, DE, USA).

To assess the relative telomere length (RTL) of the 180 subject samples, two separate quantitative polymerase chain reactions (qPCRs) were performed on the ABI Prism 7900HT Sequence Detection System, with SDS software Version 2.3 used to generate the outputs. In the first reaction, we assayed the telomere repeat region (TTAGGG). In the second qPCR, we assayed a single copy gene (albumin), which we used as an internal control to correct for minor differences in DNA concentration across samples (Cawthon et al., 2009). Both reactions were performed across 384 well plates with identical sample/well positions used. To detect for any DNA contamination, each plate contained four negative controls (RNase-free water) in triplicate. An eight-point genomic DNA (human leukocyte) dilution series (0.47 ng, 0.94 ng, 1.88 ng, 3.75 ng, 7.5 ng, 15 ng, 30 ng, 60 ng) was also included on every plate allowing for absolute quantification of each sample, and accounting for any differences in PCR efficiency between the telomere and albumin reactions. Inter-plate variability was controlled for using five calibrator samples included on each plate, consisting of leukocyte DNA from five additional human samples, and run in triplicate. To further reduce variability between runs, the calibrators and standard curve DNA (highest standard) were both prepared in one batch and aliquoted before being frozen at −20 °C. Both the calibrators and standard curve aliquots were thawed on the day of each run, with the dilution series prepared fresh on the day.

An adapted version of a qPCR protocol described in a previous paper by Cawthon was used for each reaction (Cawthon et al., 2009). Each qPCR mix for the telomere reactions consisted of 10.5  μL of 2x qPCR Mastermix with SYBR green (Primer Design, Southampton, UK), 4.5  μL of RNase free water, 12 ng of DNA, 1000 nM of telg, 5′-ACACTAAGGTTTGGGTTTGGGTTTGGGTTTGGGTTAGTGT-3′ and 800 nM of telc, 5′-TGTTAGGTATCCCTATCCCTATCCCTATCCCTATCCCTAACA-3′. Four stages made up the thermocycling conditions as follows: Stage 1: 95 °C for 15 min, Stage 2: 2 cycles for 15 s at 94 °C and 49 °C, Stage 3: 25 cycles at 94 °C for 15 s, 10 s at 62 °C, and 15 s at 73 °C (data collection), Stage 4: dissociation curve (primer specificity detection).

The same reagents and quantities as the telomere qPCR mix were used for the albumin reactions with the exception of the forward and reverse primers that were replaced with primers for the albumin gene. The quantity of both the forward and reverse primers was adjusted for the albumin reaction to 765 nM of forward primer (albu): 5′-CGGCGGCGGGCGGCGCGGGCTGGGCGGAAATGCTGCACAGAATCCTT-3′ and 930 nM of reverse primer (albd): 5′-GCCCGGCCCGCCGCGCCCGTCCCGCCGGAAAAGCATGGTCGCCTGTT-3′. The thermocycling conditions for the albumin reaction also consisted of four stages as follows: Stage 1: 95 °C for 15 min, Stage 2: 2 cycles for 15 s at 94 °C and 49 °C, Stage 3: 33 cycles at 94 °C for 15 s, 10 s at 62 °C, and 15 s at 88 °C (data collection), Stage 4: dissociation curve (primer specificity detection).

### 2.5 Statistical analysis

#### 2.5.1 Calculating relative telomere length

A standard deviation of less than 0.5 was required for at least two of the three cycle threshold (C_*t*_) technical triplicates for a sample to be included in downstream analysis. C_*q*_ values were then created from the remaining C_*t*_ values by relating them to absolute quantities as part of a standard curve. To adjust for inter-plate variability, mean adjusted C_*q*_ values were then created by dividing the mean C_*q*_ of each sample by the mean C_*q*_ of the five calibrator samples (present on each plate). The RTL was then calculated by dividing each sample's mean adjusted C_*q*_ value from the telomere reaction by each sample's mean adjusted C_*q*_ values from the albumin reaction. RTL was then log-transformed to allow for parametric analysis. Outliers were categorised as those data points greater than two standard deviations from the mean. As a final check, we performed a one-tailed Pearson correlation test to check whether there was a negative correlation between log(RTL) and age.

#### 2.5.2 Depression and maltreatment main effects analysis

To assess the main effects of case/control status and to tease apart whether childhood maltreatment or depressive disorder caseness drives differences in RTL we performed seven linear regressions. In the first univariate linear model (model 1) we selected log(RTL) as the dependent variable, and depression case-control status as the independent variable, covarying for age, gender, and study. Subsequently, we performed the same model as above in a further six regressions (models 2–7), but with the addition of maltreatment status which was dichotomised into maltreatment/no maltreatment for each of the five subtypes and a mean maltreatment category.

#### 2.5.3 Severity analyses

To determine if more severe depression was specifically associated with RTL, we repeated the first univariate model described above (model 1), but only in cases who had experienced a moderate-severe depressive episode versus controls. Furthermore, for any significant maltreatment subtypes associated with RTL, we further considered the effects of maltreatment severity (0=no maltreatment, 1=mild maltreatment, 2=moderate-severe maltreatment) on RTL in the context of depression case/control status, using the same models as described in (ii). For any significant results in this analysis, we subsequently repeated the same statistical test, but we split our sample into subject groups based on age; those in the age range of 20 and 50 (n=92), and those in the age range of age of 51 and 84 (n=88), in order to test whether the mechanisms involved in RTL erosion produce more detrimental effects over the years in older individuals (i.e. over a longer time frame).

#### 2.5.4 Depression case/control status by maltreatment interactions

For interaction analyses we performed a univariate linear model with log(RTL) as the dependent variable, depression case-control x maltreatment interaction term as the independent variable, covarying for depression case-control status, maltreatment/no maltreatment, age, gender, and study. We performed this six times for each of our dichotomised maltreatment/no maltreatment subtypes, and one mean maltreatment score.

#### 2.5.5 Multiple testing correction

We used the false discovery rate (FDR) of multiple testing correction (Benjamini and Hochberg, 1995) and set a significance threshold of q<0.05. This was conducted using an online FDR calculator (http://www.sdmproject.com/utilities/?show=FDR). We applied the FDR-correction to depression case/control main effect results, maltreatment main effect results, and results from our interaction analyses, separately, as they correspond to independent hypotheses tested multiple times.

#### 2.5.6 Sensitivity analyses

We performed a series of sensitivity analyses to ensure there were no confounding influences from BMI, subtypes of depression, physical illnesses, medications and health behaviours, see Supplementary Information.

All analyses, with the exception of FDR multiple testing corrections, were conducted using SPSS Version 22 (IBM, New York, USA), with plots generated using GraphPad (GraphPad Software Inc, California, USA).

## 3 Results

### 3.1 Quality control checks

Standard curves from all reactions revealed an R^2^≥0.99 between quantity of known DNA and C_*t*_ values. No amplification was detected in the negative controls present on any plate. A single clear peak was observed across dissociation curves (melting curves), indicating that specificity of amplification was achieved throughout all plates, see Supplementary Information. The telomere reaction achieved a mean PCR efficiency of 87.1%, and the albumin reaction had an efficiency of 79.1%, which is consistent with previously published results from studies using qPCR to assay RTL (Hovatta et al., 2012), and corrected for through the use of a standard curve. All samples passed our quality control criteria and none were identified as outliers.

### 3.2 Telomere length and chronological age

**Fig. 1:**
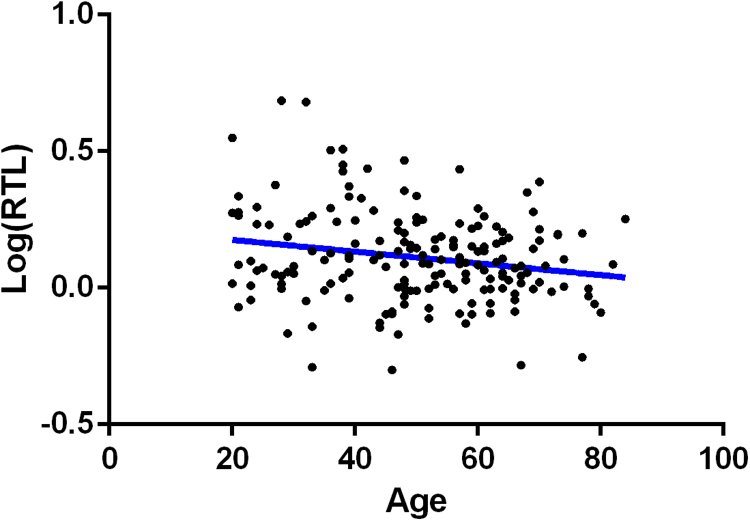
A scatterplot showing a negative correlation between chronological age, x-axis, and log(RTL), y-axis. Line represents the line of best fit.

A Pearson's correlation revealed that RTL was negatively correlated with age, r(178)=−0.259, p=2.290E-04,(Fig. 1) as expected.

### 3.3 Depression and maltreatment main effects analysis

Emotional neglect was the most frequently reported subtype of childhood maltreatment (cases=44; controls=36), followed by emotional abuse (cases=38; controls=22), with sexual abuse being the least reported form of childhood maltreatment (cases=21; controls=14), Table 2.

**Table 2:** Frequency table showing total number of both cases and controls, in relation to each maltreatment subtype.

		**Nō of cases**	**Nō of controls**
Case/Control status		80	100
Mean abuse	**No**	41	81
	**Yes**	39	19
Physical abuse	**No**	56	88
	**Yes**	24	12
Emotional abuse	**No**	42	78
	**Yes**	38	22
Sexual abuse	**No**	59	86
	**Yes**	21	14
Physical neglect	**No**	42	84
	**Yes**	38	16
Emotional neglect	**No**	36	64
	**Yes**	44	36

**Fig. 2:**
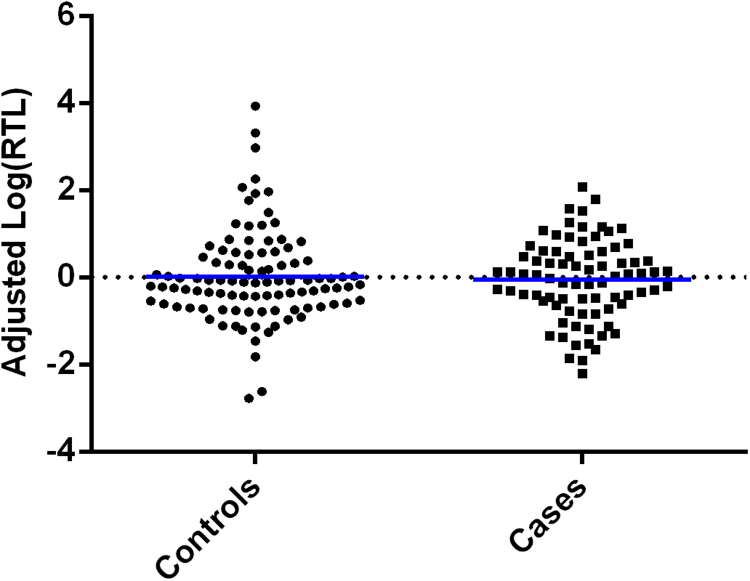
A plot showing log(RTL) adjusted for the effects of age, sex and study (*y*-axis), in depression cases and controls (*x*-axis). There were no differences found between cases and controls.

**Fig. 3:**
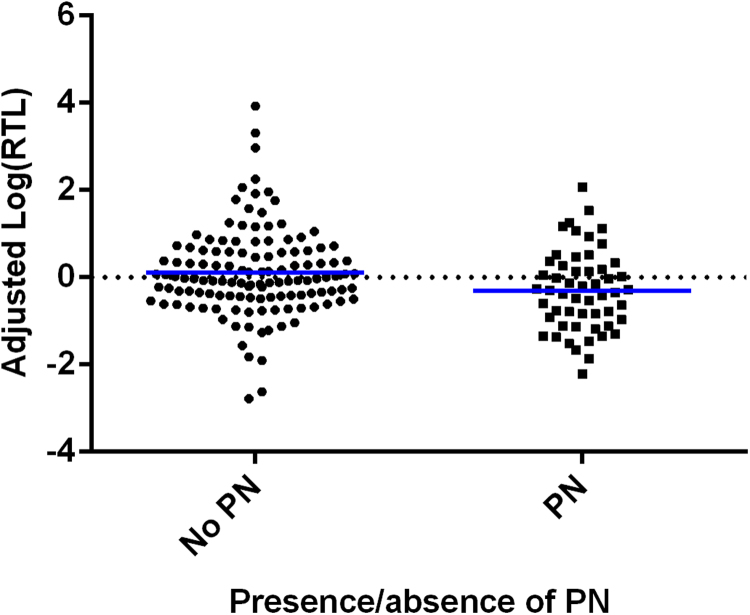
A plot showing log(RTL) adjusted for the effects of age, sex and study (*y*-axis) in those who have and have not experienced physical neglect (PN; x-axis). There was a significant decrease in adjusted log(RTL) amongst those who had experienced childhood physical neglect (p=0.007).

Linear regressions revealed no effect of depression case/control status on RTL, and this remained non-significant after covarying for maltreatment subtypes, Table 3, Fig. 2. Similarly, there was no effect of mean maltreatment, or four of the maltreatment subtypes, on RTL, Table 3. However, childhood physical neglect did show a significant association with shortened RTL (F(1, 174)=7.559, p=0.007, q=0.042, Variance Explained=4.2%), Fig. 3.

**Table 3:** Table showing the F values, P-values, and false discovery rate corrected Q-values from univariate linear regressions investigating the effects of depression status on log(RTL), model 1; and subsequently the effects of depression status whilst covarying for childhood maltreatment subtypes, models 2–7. Age, sex and study were also included as covariates in all analyses.

**Model**	**Variable**	**F**	**p-value**	**q-value**	**Variable**	**F**	**p-value**	**q-value**
1	Case/Control status	0.351	0.554	0.894	–	–	–	–
2	Case/Control status	0.089	0.766	0.894	Mean abuse score	1.254	0.264	0.528
3	Case/Control status	0.127	0.722	0.894	Physical abuse	2.311	0.130	0.390
4	Case/Control status	0.402	0.527	0.894	Emotional abuse	0.076	0.784	0.836
7	Case/Control status	0.389	0.534	0.894	Sexual abuse	0.284	0.595	0.836
5	Case/Control status	0.001	0.979	0.979	Physical neglect	7.559	**0.007**	0.042*
6	Case/Control status	0.304	0.582	0.894	Emotional neglect	0.043	0.836	0.836

* indicates q<0.05.

### 3.4 Severity analyses

When restricting our case-control analysis to just those with moderate-severe depression relative to controls, there were still no significant differences in RTL (F(1, 150)=0.651, p=0.421, variance explained=0.4%). When we considered the severity of physical neglect as an ordinal measure (none (0), n =126); mild (1), n =29; moderate-severe (2) n=25), we found that this too predicted RTL, with the shortest RTL amongst those individuals with moderate-severe neglect (F(1, 174)=7.726, p=0.006, variance explained=4.3%). Furthermore, when we split our cohort into a younger (≤50 years old; no maltreatment, n=59; mild maltreatment, n=17, moderate-severe, n=16) and older (>50 years old; no maltreatment, n=67; mild maltreatment, n=12, moderate-severe, n=9) age subgroups, we found that RTL was only significantly shorter in the older individuals (F(1, 82)=4.871, p=0.03, variance explained=5.6%), but not the younger individuals (F(1, 85) =2.985, p=0.088, variance explained=3.4%).

### 3.5 Depression case/control status by maltreatment interactions

Analysis of the interaction between childhood maltreatment subtypes and depression case/control status revealed that there were no statistically significant interactions between any of the five childhood maltreatment subtypes and depression disease status with regards to their impact on RTL, before or after controlling for FDR (Table 4).

**Table 4:** Table showing the F values, p-values, and false discovery rate corrected q-values for interactions between childhood maltreatment subtypes and depression case/control status, as affecting log(RTL). Age, sex, and study were also included as covariates in all analyses.

**Model**	**Variables**	**F**	**p-value**	**q-values**
1	Mean abuse×Case/Control status	0.009	0.923	0.939
2	Physical abuse×Case/Control status	0.646	0.423	0.846
3	Emotional abuse×Case/Control status	1.023	0.313	0.846
4	Physical neglect×Case/Control status	0.024	0.877	0.939
5	Emotional neglect×Case/Control status	1.072	0.302	0.846
6	Sexual abuse×Case/Control status	0.006	0.939	0.939

### 3.6 Sensitivity analysis

BMI had no significant impact on log(RTL) in our sample [F(1, 172)=1.651, p=0.201]. Within-cases there was also no significant effects of the depression subtypes on log(RTL) [F(2, 74)=1.501, p=0.230]. Sensitivity analyses demonstrated that there were no significant effects of health behaviours (smoking status, drug dependency and drug use), co-existing illnesses, or use of medication on RTL (p>0.05), see Supplementary Information.

## 4 Discussion

This study aimed to investigate the impact of childhood maltreatment, depression case/control status and the interaction between the two, on RTL, a marker of biological ageing (Eitan et al., 2014). The rationale of the study was to improve our understanding of whether specific childhood maltreatment subtypes may drive shortened telomere lengths previously reported amongst depressed patients. Furthermore, we wanted to consider whether or not maltreatment impacted on RTL more pervasively amongst depressed cases, and therefore whether biological ageing may contribute to depression-related pathophysiology.

We found that depression case/control status had no significant effect on RTL, Fig. 2*,* and this remained non-significant even in the moderate/severe depressed only subgroup. Not surprisingly, as there was no main effect of depression status on RTL, once we included maltreatment subtypes as covariates, depression case/control status remained non-significant, Table 3. The absence of a case/control difference is in line with some previous research (Wolkowitz et al., 2011, Teyssier et al., 2012, Needham et al., 2015, Schaakxs et al., 2015). However, it can also be seen as adding to the divergent and collectively inconclusive findings involving the relationship between depression and RTL, as there have also been a similar, albeit a slightly higher number of previous findings, in keeping with Simon and colleagues’ original study (Simon et al., 2006), demonstrating a significant relationship between depression and shorter RTL (Garcia-Rizo et al., 2013, Elvsåshagen et al., 2011, Hoen et al., 2011, Lung et al., 2007, Verhoeven et al., 2014, Wikgren et al., 2012, Schutte and Malouff, 2015, Ridout et al., 2016, Darrow et al., 2016, Lin et al., 2016). One possible explanation is that, in at least some of the previous reports, there was an enrichment of maltreated individuals amongst the depressed cases, which may have driven the association. Indeed, our analyses revealed that the childhood maltreatment subtype, physical neglect, was specifically and singly associated with shortened RTL in adulthood, Fig. 3. It may also represent an important mechanism mediating the relationship between lower socioeconomic status in childhood, where basic physical needs cannot always be met optimally (e.g. healthcare and nutrition), and higher risk for ageing related disorders in adulthood (Guralnik et al., 2006).

Severity analyses further revealed that telomere shortening correlated with physical neglect severity, with the moderate-severely maltreated individuals having the shortest RTL. Furthermore, when splitting up our cohort by age group (≤50 or >50 years old), we found that shorter RTL was more strongly associated with physical neglect amongst the older individuals. This may suggest that physical neglect in childhood activates telomere-shortening mechanisms (e.g. excessive cortisol, inflammatory activation), with the impact being greatest amongst older individuals, where the mechanism has had a longer time to evoke its detrimental effects.

Investigating the interaction between depression case/control status and maltreatment subtypes was a novel approach, however, our study revealed no significant interactions, Table 4. This may suggest that disease-specific changes to telomere shortening in response to maltreatment, is unlikely. Furthermore, in the case of physical neglect, where we observed a significant main effect, it suggests that telomere shortening in response to physical neglect may not represent a biological mechanism important in mediating susceptibility to adulthood depression. However, we acknowledge that the relatively small sample sizes within our cohort may limit our power to detect interaction effects, so this may still be of interest in future studies.

Despite the interesting findings detailed in this study, it's important to consider its limitations. Although our study had a moderately large sample size, especially relative to other previous reports, e.g. Tyrka and colleagues’ study comprising of 31 individuals (Tyrka et al., 2010), it may still be underpowered. Furthermore, low numbers of subjects who suffered from moderate or severe maltreatment subtypes, meant we had to dichotomise maltreatment, at least for our primary analysis. Although we could consider severity for physical neglect as a secondary analysis, we were underpowered to investigate the effects of severity for other maltreatment subtypes (e.g. sexual abuse). The absence of further biological data such as telomerase activity and inflammation data is another limitation, as it prevented us from determining mechanisms which might underlie the association between physical neglect (and severity) and RTL, and this should be considered in future studies.

We were also unable to consider the effects of age-of-onset, chronicity, or recurrence, all of which may impact upon RTL (Schaakxs et al., 2015). Furthermore, a longitudinal study design may have avoided any possible retrospective recall biases on maltreatment. A longitudinal study design with multiple DNA samples collected over time, would also aid in confirming the temporal ordering of events such as maltreatment and depression-onset, and their subsequent impact on RTL as an individual ages. Nevertheless, we believe the current study has good reliability due to the stringent controls implemented, including quality control of the telomere assessment; sensitivity analyses to check for the effects of confounding health behaviours, medication use and comorbid illnesses; as well as multiple testing correction.

To conclude, our study suggests that physical neglect specifically contributes to shortened RTLs. The impact of physical neglect, however, was independent from depression case/control status. Furthermore, as physical neglect is a risk factor for depression and tends to be more frequent amongst depressed cases, it represents one potential confounder driving previous associations between depression and shorter RTL.

### 4.1 Funding source

TRP is funded by a Medical Research Council Skills Development Fellowship (MR/N014863/1), and the current project was funded by a Psychiatry Research Trust Grant awarded to TRP and GB. DeCC was funded by the Medical Research Council, UK. SELCoH was supported by the Biomedical Research Nucleus data management and informatics facility at South London and Maudsley NHS Foundation Trust, which is funded by the National Institute for Health Research (NIHR) Mental Health Biomedical Research Centre at South London and Maudsley NHS Foundation Trust and King's College London and a joint infrastructure grant from Guy's and St Thomas’ Charity and the Maudsley Charity. Phase 3 of the SELCoH study was also funded by the Maudsley Charity. MH, SLH, SF, LG and GB are supported by the National Institute for Health Research (NIHR) Mental Health Biomedical Research Centre, South London and Maudsley NHS Foundation Trust and King's College London. The views expressed are those of the authors and not necessarily those of the NHS, the NIHR or the Department of Health. The funding sources had no role in the study the design, in the collection, analysis, and interpretation of data, in the writing of the report and in the decision to submit the article for publication.
