# Supplementary material for: Assessing the contributions of childhood maltreatment subtypes and depression case-control status on telomere length reveals a specific role of physical neglect
Source: J Affect Disord. 2017 Apr 15;213:16–22. doi: 10.1016/j.jad.2017.01.031 (PMC6191534; doi:10.1016/j.jad.2017.01.031)
Supplement: Supplementary file 1 — Supplementary material [file mmc1.docx]

**Supplementary Information**

**S1: Sensitivity Analyses**

To ensure BMI wasn’t significantly affecting log(RTL) in our sample, we performed a linear regression, with log(RTL) as the dependent variable, BMI as the independent variable, covarying for age, sex and study.

We also performed sensitivity analyses to investigate if there were significant differences in log(RTL) between our mild depressive disorder, moderate depressive disorder cases, severe depressive disorder cases, and mixed mild depression and anxiety cases. To achieve this, we performed a within-case analysis to investigate if these factors predicted log(RTL), covarying for age, gender and study.

Within the SELCoH depression cases we also checked for the influences of physical illnesses, medications and health behaviours on log(RTL), as rates of comorbid illnesses, medication use, and poor health behaviours (e.g. smoking) can be higher amongst depressed cases, and may drive associations. Univariate linear regressions were run for all data regarding life style habits (smoking status, drug dependency and drug use), physical illnesses and medication use, against log(RTL), covarying for age and gender.

**S2: Dissociation Curves**


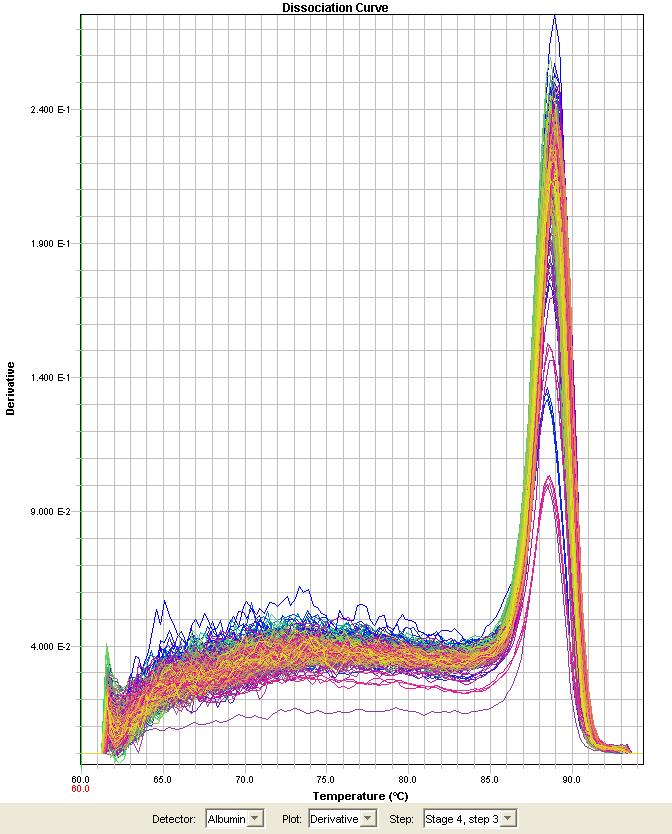

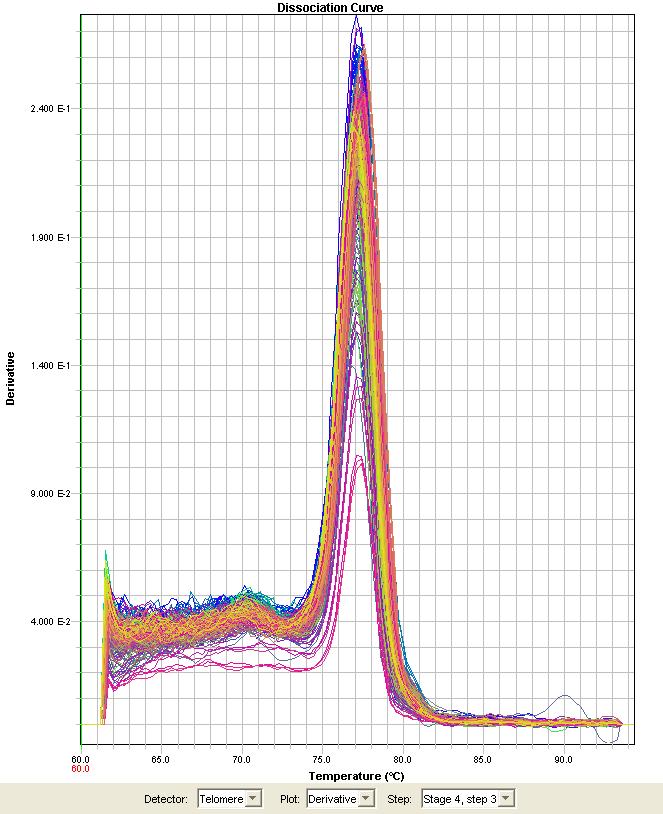


**S2:** Dissociation curves showing specific amplification for both the telomere repeat region (left) and albumin single copy gene (right), both produced during the qPCR reactions; obtained from ABI Prism SDS Software version 2.2 output.

**S3: Results from sensitivity analyses investigating the effects of potential confounders on log(RTL)**

**Smoking and Drug Use**

| **Smoking and Drugs** | **F** | **d.f.** | **p-values** | **Effect Size** |
| --- | --- | --- | --- | --- |
| Smoking Status | .266 | 1 | .607 | .003 |
| Drug Dependency | 1.351 | 1 | .248 | .013 |
| Drug Use | 3.520 | 1 | .063 | .032 |

**Physical Illnesses**

| **Illnesses** | **F** | **d.f.** | **p-values** | **Effect Size** |
| --- | --- | --- | --- | --- |
| Asthma | .209 | 1 | .648 | .002 |
| Depression/other Nervous Illnesses | .000 | 1 | .992 | .000 |
| Diabetes | .107 | 1 | .744 | .001 |
| Stomach/Digestive Disorders | 3.378 | 1 | .069 | .031 |
| Rheumatic Disorders/Arthritis | .003 | 1 | .958 | .000 |
| Heart Trouble | .796 | 1 | .374 | .008 |
| Stroke | .033 | 1 | .857 | .000 |
| High Blood Pressure | .040 | 1 | .842 | .000 |
| Migraines | .763 | 1 | .384 | .007 |
| Epilepsy | .442 | 1 | .508 | .004 |
| Gynaecological Problems | .001 | 1 | .978 | .000 |
| Cancer | 1.593 | 1 | .210 | .015 |
| Kidney Problems | .023 | 1 | .881 | .000 |
| Other | .001 | 1 | .972 | .000 |
| Long Lasting Illnesses | .383 | 1 | .537 | .004 |
| Number of Long Lasting Illnesses | .389 | 1 | .534 | .004 |

**Medications and Supplements**

| **Medications** | **F** | **d.f.** | **p-values** | **Effect Size** |
| --- | --- | --- | --- | --- |
| Pain medication | 1.687 | 1 | .197 | .016 |
| Antacid Medication | .404 | 1 | .527 | .004 |
| Cold Medication | .024 | 1 | .878 | .000 |
| Allergy Medication | 1.741 | 1 | .190 | .016 |
| Antibiotic Medication | .003 | 1 | .953 | .000 |
| Birth Control Medication | .424 | 1 | .516 | .004 |
| Chest Medication | .940 | 1 | .335 | .009 |
| Diabetes Medication | .107 | 1 | .744 | .001 |
| Depression/Anxiety Medication | .001 | 1 | .976 | .000 |
| Heart/blood pressure Medication | .011 | 1 | .915 | .000 |
| Thyroid Medication | .840 | 1 | .362 | .008 |
| Other Medication | 1.932 | 1 | .168 | .018 |
| Vitamin Supplements | .309 | 1 | .580 | .003 |
| Herbal Medication | .157 | 1 | .693 | .001 |
| Any Medication | .558 | 1 | .457 | .005 |

**S3**: All results are based on univariate linear regressions which included age, sex and BMI as covariates, and log(RTL) as the outcome variable.
